# Pulse Dosing of 10-kHz Paresthesia-Independent Spinal Cord Stimulation Provides the Same Efficacy with Substantial Reduction of Device Recharge Time

**DOI:** 10.1093/pm/pnab288

**Published:** 2021-10-02

**Authors:** David Provenzano, Jordan Tate, Mayank Gupta, Cong Yu, Paul Verrills, Maged Guirguis, Nathan Harrison, Thomas Smith, Rose Azalde, Kerry Bradley

**Affiliations:** 1 Pain Diagnostics & Interventional Care, Sewickley, Pennsylvania, USA; 2 Alliance Spine & Pain Centers, Atlanta, Georgia, USA; 3 Neuroscience Research Center, Overland Park, Kansas, USA; 4 Swedish Medical Center, Seattle, Washington, USA; 5 Metro Pain Group, Clayton, VIC, Australia; 6 Ochsner Medical Center, New Orleans, Louisiana, USA; 7 Guy’s and St. Thomas’ Hospital NHS Trust, London, UK; 8 Nevro Corporation, Redwood City, California, USA

**Keywords:** Spinal Cord Stimulation, High-Frequency Stimulation, 10 kHz, Chronic Low Back Pain, Pulse Dosing, Duty Cycling

## Abstract

**Objective:**

This study was designed to assess whether using pulse dosing (PD) (regularly cycled intermittent stimulation) of high-frequency 10-kHz spinal cord stimulation (10-kHz SCS) can reduce device recharge time while maintaining efficacy in patients with chronic intractable back pain with or without leg pain.

**Design:**

Prospective, multicenter, observational study.

**Methods:**

Patients successfully using 10-kHz SCS at 100%ON (i.e., continuously with no PD) for >3 months were consecutively enrolled. After a 1-week baseline period of documenting their pain twice daily on a 0–10 numerical rating scale (NRS) using 100%ON of their “favorite” program, all subjects were reprogrammed to 14%PD for 10–14 days. If subjects preferred 14%PD to 100%ON, they were programmed to 3%PD; otherwise, they were programmed to 50%PD. Subjects used this next program for another 10–14 days. Subjects then entered a 3-month observational period during which they were requested to use but not limited to their most preferred %PD program. Toward the end of 3 months, subjects completed a 7-day NRS diary and indicated a final %PD program preference. Study endpoints included %PD preference, mean diary NRS by %PD, and daily minutes and patterns of charging.

**Results:**

Of 31 subjects completing the study, 81% preferred less than 100%ON. Among the subjects, 39% preferred 3%PD, 32% preferred 14%PD, 10% preferred 50%PD, and 19% preferred 100%ON. Average daily charge durations were 8.3 ± 3.1 minutes for 3%PD, 13.9 ± 4.9 minutes for 14%PD, 26.2 ± 7.4 minutes for 50%PD, and 43.8 ± 10.9 minutes for 100%ON. Regression modeling suggested that pain relief was weighted as more than twice as influential as charging in preference for reduced %PD.

**Conclusions:**

This prospective study suggests that 10-kHz SCS therapy with PD may be successfully used in a large majority of 10-kHz SCS responders, maintaining efficacy while reducing device charging time by nearly two thirds.

## Background

Spinal cord stimulation (SCS) is currently indicated as a treatment for chronic intractable pain of the trunk and limbs [1]. Early SCS devices were externally powered via radiofrequency coupling. These devices could provide high levels of stimulation power but were burdened by requiring the external transmitter be in close proximity to the implanted receiver, a significant inconvenience for patients. In 1980, borrowing from rapidly advancing pacemaker technology, neurostimulators powered by primary cell batteries were introduced [2]. These devices had fully contained power sources, allowing patients to go about daily living unencumbered by an external device. However, the longevity of these devices was limited, requiring surgical replacement every few years even when low-energy stimulation programs were used [3].

In 2004, the first rechargeable spinal cord stimulators were introduced, which used lithium ion battery technology. Obvious benefits of these systems were small size and an extended system lifetime [4]. However, a key benefit of rechargeable systems was the enablement of new stimulation strategies, where high stimulation power could be delivered without concern for frequent implantable pulse generator (IPG) replacement or the need to wear an external power source to provide therapy. One method, using a high-frequency 10-kHz pulse rate, was demonstrated by Kapural et al. as statistically and clinically superior to traditional, low-frequency SCS in relief of predominant back and radicular leg pain [5, 6].

Still, although patients using 10-kHz SCS have indicated that their daily recharge time is quite manageable, the ability to reduce the needed daily amount of recharging would likely be welcomed if efficacy could be maintained [7–9]. In the aforementioned Kapural et al. study, some subjects were offered pulse dosing (PD) of 10-kHz SCS, both as an exploratory mode of stimulation delivery as well as a possible technique to reduce the device recharging time. In a PD mode, the stimulation is cycled between brief states of “ON” and “OFF,” e.g., at 14%PD, pulses are delivered at 10 kHz for 20 seconds (“ON”), then no pulses are delivered for 2 minutes (“OFF”), and then the cycle repeats. This means that less overall stimulation charge is delivered and the drain on the rechargeable battery is reduced, allowing for reduced recharging time. Although not a studied outcome, many patients seemed to respond to the PD stimulation; the most commonly used PD setting in the Kapural et al. study was 14%PD (20 seconds ON and 2 minutes OFF). The purpose of the present prospective study was to determine whether PD of high-frequency 10-kHz SCS can maintain pain reduction while reducing device recharge time.

## Methods

This study was conducted at five centers in the United States and one center in Australia. All centers obtained investigational review board or ethics committee approvals (as appropriate to country of site), and all subjects provided informed consent. The study was conducted in accordance with local clinical research and data protection regulations, good clinical practice guidelines (ISO 14155), and the Declaration of Helsinki. This study was registered on March 1, 2016 (ISRCTN54708653), before the first subject enrollment.

### Device Description

The rechargeable Senza^®^ SCS system (Nevro Corp., Redwood City, CA, USA) received the CE Mark in 2010, Australian Therapeutic Goods Administration approval in 2011, and U.S. Food and Drug Administration approval in 2015 for use in the management of chronic intractable pain of the trunk and/or limbs. This system delivers electrical stimulation to the spinal cord through the use of a fully implantable pulse generator (IPG) and epidural leads, which carry 8–16 platinum iridium electrodes. Although this system can deliver stimulation frequencies from 2 Hz to 10 kHz, in the present study, only paresthesia-independent 10-kHz stimulation was administered.

### Patient Selection

To be enrolled in this study, patients had to meet at least the key inclusion criteria and to not meet the exclusion criteria in Table 1.

**Table 1.** Inclusion and exclusion criteria for enrollment

Key Inclusion Criteria	Key Exclusion Criteria
Diagnosed with chronic, intractable back pain with or without leg pain secondary to failed back surgery syndrome (FBSS). Implanted with the Nevro Senza SCS system with dual leads, approximately over vertebral T8–T11, for at least 3 months, and are using the system with a single contact combination, continuous 10-kHz stimulation programs for at least 18 hours daily for a minimum of 21 days before enrollment. Stable chronic pain medications. 18 years of age or older. Compliant in using the patient programmer and recharger as determined by the investigator. Considering daily activity and rest, report a recall average back pain relief of >50% compared with before implantation and a recall average NRS score for back pain of <5 during the previous 14 days before study enrollment.	Cannot have a medical condition or pain in other area(s), not intended to be treated with SCS, that could impact study outcome assessments. Cannot have evidence of an active disruptive psychological or psychiatric disorder or other known condition significant enough to impact study outcome assessments. Cannot have undergone an interventional procedure and/or surgery to treat back or leg pain other than Senza HF10 therapy in the prior 30 days. Cannot be pregnant or planning to become pregnant during the course of the study. Cannot have another active implantable medical device.

### Procedures

Study candidates were identified from existing patients who were using 10-kHz SCS at each clinical site. These patients were consecutively screened, and those who signed the informed consent underwent evaluations to determine eligibility for the study on the basis of the inclusion and exclusion criteria. Subjects meeting all the inclusion criteria and none of the exclusion criteria were enrolled in the study.

Enrolled subjects then participated a baseline/run-in assessment, in which the subject-identified “favorite” preexisting 100%ON program used. Subjects used this program for approximately 1 week, with stimulation amplitude adjustments as needed, while documenting their pain relief in a twice-daily pain diary. Subjects then returned to the clinic for diary collection and underwent post-enrollment baseline/run-in screening (which included measures of diary completion compliance, stimulator system use, and pain relief). Subjects who met baseline/run-in screening criteria then moved to the next phase of the study, the “pulse dose selection period.”

Each subject’s run-in 100%ON program was then converted to a 14%PD program. Subjects left the clinic for a 10- to 14-day period, the final 7 days of which were used for program assessment (i.e., estimation of pain relief in a twice-daily pain diary). During this 14%PD period, subjects were guided to alter their program amplitudes as needed to achieve good pain relief.

After the 14%PD program assessment period, subjects were asked whether they preferred the 14%PD program over the previous 100%ON program. Those expressing preference for the 14%PD program or expressing no preference had their 14%PD program converted to a 3%PD program (20 seconds ON and 10 minutes OFF). Other subjects expressing preference for the 100%DC program had their 14%PD program converted to a 50%PD program (20 seconds ON and 20 seconds OFF).

All subjects then used this second PD program for another 10- to 14-day period, the final 7 days of which were used for program assessment (estimation of pain relief in a twice-daily pain diary). Again, during this period, subjects were guided to alter their program amplitudes as needed to achieve good pain relief.

After this second PD program assessment period had elapsed, the subjects entered a 3-month uncontrolled observational period. During this phase, the subjects generally received three programs: their baseline/run-in 100%ON program and their first- and second-most preferred PD programs. The subject was requested to use the study %PD program they preferred most but was allowed to choose any of the three programs at any time. If the subject requested reprogramming during the observational period, it was provided to optimize the subject’s desired outcomes, independent of the outcomes of the PD assessments. Approximately 10–14 days before the end of the observational period, each subject completed a twice-daily pain diary for 7 days, which was used for estimation of pain relief for the current active program. After the third month of the observational period and final diary completion, the subject completed participation in the study.

**Figure 1.**
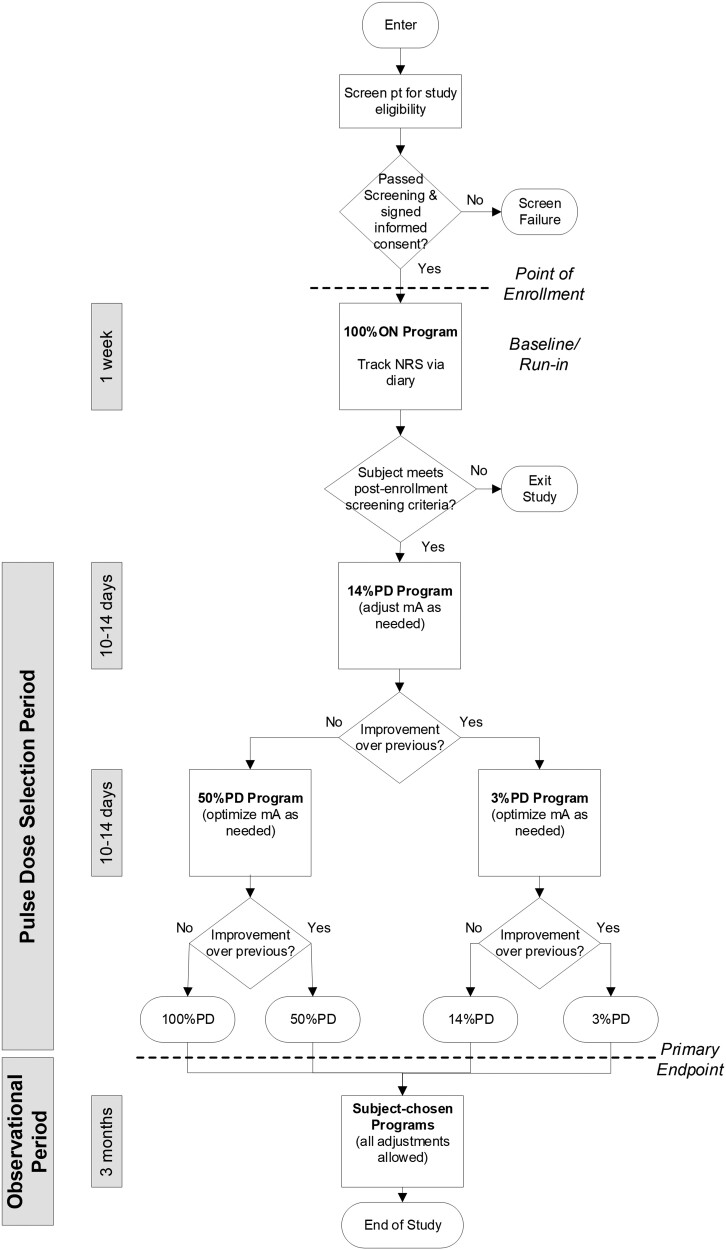
Study flow.

Figure 1 summarizes the sequence of study-related assessments, procedures, and activities for the study.

### Data Collection and Statistical Analysis

At each clinic visit, subjects returned completed pain diaries and received new ones for the next phase of the study. Additionally, at most clinic visits, subjects completed questionnaires on program preference, satisfaction, global impression of change, medication use, and reported program and device use. Also, device parameters and program usage logs were uploaded from the IPG and stored in a file for later analysis. Adverse events (AEs) were queried throughout the study and addressed as needed.

Descriptive statistics were calculated for each analyzed variable, including the number of observations, proportions, mean, median, and standard deviation. Two-tailed paired *t*-tests were used to analyze continuous variables, such as the numerical rating scale (NRS) (considered continuous for the purposes of this analysis). The normality and symmetry of the data were evaluated by the Shapiro-Wilk test, and where appropriate, parametric (e.g., analysis of variance [ANOVA]) and nonparametric (e.g., Kruskal-Wallis, Mann-Whitney) methods were used. AEs are reported descriptively for all patients. A *P* value less than or equal to 5% (*P* < 0.05) was considered to be statistically significant.

The primary endpoint was preference for any PD other than a 100%ON stimulator program (treatment) or no preference, vs preference for the 100%ON stimulator program (control). From the preference ratings, “PD responders” were defined as those expressing preference for the PD program, and nonresponders were subjects who preferred 100%ON stimulation programs. PD responder and nonresponder rates were analyzed with Fisher’s exact test; although there was no a priori statistical hypothesis, the expected response was that 67% of subjects would choose <100%ON, as this was the anecdotally observed rate of successful use of PD in the SENZA randomized controlled trial [5].

Satisfaction and Patient Global Impression of Change (PGIC) were analyzed by forming groups of PD responders (<100%ON) and PD nonresponders (100%ON) and analyzing dichotomously grouped outcomes: for the satisfaction outcome, the groups were “at least satisfied” or “not sure/dissatisfied/very dissatisfied”; for PGIC, the groups were “at least improved or no change” or “worse.” Similarly, to assess whether PD responders were still content with using <100%ON programs, for the same groupings, satisfaction and PGIC were compared between baseline/run-in and the end of the observational period. Both satisfaction and PGIC were analyzed with Fisher’s exact test.

For continuous variables, subjects were categorized into one of four %PD groups, depending on their most preferred %PD: 100%ON, 50%PD, 14%PD, and 3%PD. Back pain intensity and leg pain intensity, averaged from diary entries, were analyzed with single-factor ANOVA.

Log data from the IPG were used to confirm program usage throughout the study. If IPG log data for the pulse dose selection period or observational period were not available, no assumption was made about program use. The program that the subject used for the majority (>50%) of the time during the observational period was used for categorization and responder analyses.

Log data from the IPG were also used to determine device charging metrics. Averaged daily charge time was compared across %PD groups with single-factor Kruskal-Wallis. Also, charging patterns (i.e., days between device recharging sessions) were analyzed to determine how subjects’ device-charging behavior might change with PD.

The primary endpoint of the study was program preference. The use of PD allowed the subjects in this study to assess program preference with generally shorter device recharge times than they had experienced before entry. Thus, the reasons for preferring one program vs another could include several factors. To gain insight into the relative importance of therapeutic efficacy vs device recharging, after the 14%PD assessment period, subjects rated the strength of their preference (or nonpreference) for the 14%PD program vs the 100%ON program in three predictor variables: pain relief, device charging, and “stimulation experience.” For each of these three factors, subjects could weight how much they preferred the just-used 14%PD vs the previously used 100%ON programs, by selecting: “strongly prefer present program” (i.e., the 14%PD program), “somewhat prefer present program,” “no preference,” “somewhat prefer previous program” (i.e., the 100%ON program), and “strongly prefer previous program.” Program preference itself had three levels (1–3), where the highest level (level 3) represented a preference for 14%PD, level 2 reprsented no preference, and level 1 represented a preference for 100%ON. A multiple regression model was constructed with “program preference” as the output and the weighted factors above as input variables.

## Results

### Enrollment, Demographics, and AEs

Forty-two (42) subjects signed informed consent forms and were enrolled. Ten subjects did not meet inclusion/exclusion criteria, and one failed the post-enrollment baseline/run-in screening criteria. Thus, thirty-one (31) subjects met all inclusion and no exclusion criteria, passed the post-enrollment baseline/run-in screening, and completed the study. Table 2 shows the demographics for these subjects.

**Table 2.** Demographics of study subjects

Characteristic	Value
Gender, M/F, n	17/14
Age, y	65 ± 13
Weight, kg	96 ± 22
Height, cm	171 ± 11
Pain duration, y*	7.5 ± 6
Implant duration before study entry, y	1.1 ± 0.7
Pain scores at study entry:	
Back pain	2.6 ± 1.7
Leg pain	1.7 ± 1.9
Pain relief at study entry:	
Back pain	80 ± 16%
Leg pain	70 ± 32%
Distribution of leg pain, number of subjects reporting	
Unilateral	15
Bilateral	10
None	5
Diagnoses, number of subjects reporting (subject could not have more than one)	
Radiculopathy	19
Mild/moderate spinal stenosis	15
Degenerative disc disease	12
Spondylosis	8
Neuropathic pain	8
Spondylolisthesis	4
Sacroiliac dysfunction	2
Other chronic pain	10

* Pain duration is defined as the time elapsed between the date of the initial diagnosis of chronic pain and the date the patient received the SENZA IPG.

As shown in Table 3, there were 11 AEs in 8 subjects. Eight of these were mild (muscular/sensory phenomena, such as spasm or pruritis; secondary to other medical conditions, such as increased activity, motor vehicle accident, preexisting cardiac conditions). One was moderate (increased back pain secondary to motor vehicle accident). These nine were mostly resolved without intervention or addressed successfully with medication adjustments. Two were severe: One was post-thoracotomy pain that was addressed with surgery; the other was breakthrough pain of the lower extremities, attributed to inability to receive scheduled injections during the study because of COVID-19 challenges. One AE was attributed to the device (perceived paresthesia, actually due to a preexisting neurological disorder and not causing distress). Two were serious, requiring hospitalization: the aforementioned post-thoracotomy pain and the other for treatment and observation after collapse from inappropriate self-dosing of tinazidine. Both were resolved without study withdrawal and were unrelated to the study or device.

**Table 3.** Adverse events

Total AEs, n	11
Subjects with an AE, n	8
AEs by type	
Serious AE	2
Nonserious AE	9
Number of unanticipated adverse device effects^a^	0
AEs by relationship to study (procedure, device, or stimulation)	
Not related	10
Study-related events	1
Device-related events	0
Procedure-related events	0
Stimulation-related events	1
AEs by severity	
Mild	8
Moderate	1
Severe	2
AEs by outcome	
Resolved	9
Ongoing^b^	2

a An unanticipated adverse device effect is defined as an event that is unanticipated in nature (e.g., is not predefined in the protocol); is device, procedure, or stimulation related; and is serious.

b One AE was increased back pain secondary to motor vehicle accident during study; the other AE was intermittent pain (not intended for treatment by SCS) that required periodic steroid injections, which subject could not obtain because of COVID-19.

### Primary Endpoint

**Figure 2.**
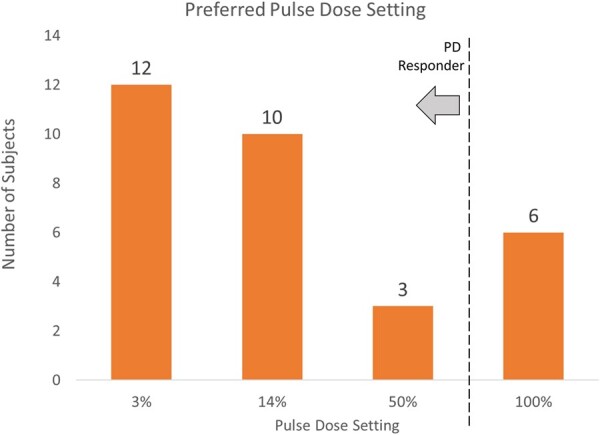
Distribution of initial preference of %PD setting.

In the overall pulse dose selection period, 25 of the 31 subjects (81%; 95% confidence interval: 62.5% to 92.5%) preferred any PD vs <100%ON. This was statistically equivalent to the a priori expectation that 67% of subjects would prefer PD (*P* < 0.001; Fisher’s exact test). Our observed 81% responder rate was also significantly greater than the 19% (6/31) who preferred the 100%ON setting. After the first test of 14%PD, 71% (22/31) preferred it over 100%ON (*P* = 0.002). When 3%PD was tested next, the preference between 3%PD and 14%PD was split, with 55% of subjects preferring 3%PD and 45% preferring 14%PD. In the nine who received the 50%PD, three preferred it over 100%ON. There were no subjects who expressed no preference. Figure 2 shows the breakdown of PD responders by %PD after the pulse dose selection period and the start of the observational period.

Tracking of stimulation program use through the observational period was available for 27 of the 31 subjects (three subjects were followed up remotely without access to IPG memory; one subject had file mis-synchronization). Of these, 23 subjects (85%) continued to use their initially preferred %PD, either exclusively or for the majority (84%; 95% confidence interval: 71% to 97%) of the observational period. Of all subjects, six changed from their initially preferred %PD during the observational period: Four subjects reverted to 100%ON, and two subjects chose a lower %PD than initially preferred.

### Pain Intensity, Satisfaction, and Global Impression of Change

**Figure 3.**
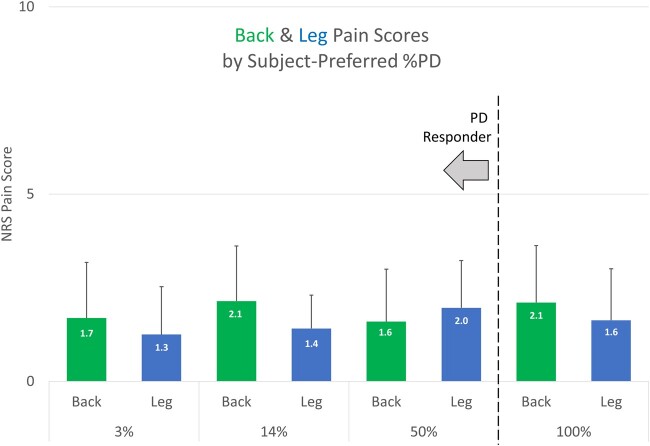
Distribution of back pain intensity and leg pain intensity scores by subject-preferred %PD.

Figure 3 shows the mean back and leg pain intensity by subject-preferred %PD for all subjects during the pulse dose selection period. Neither back pain nor leg pain intensity was statistically related to %PD group, as determined by one-way ANOVA (back: *F*(3,26) = 0.32, *P* = 0.81; leg: *F*(3,21) = 0.26, *P* = 0.85). Overall, back pain intensity and leg pain intensity were not significantly changed from the 100%ON baseline, suggesting that most responding subjects can use their most preferred %PD without loss of efficacy. Indeed, there was no significant difference in the NRS back pain scores between the pulse dose selection period and the observational period for PD responders (*P* = 0.11).

**Figure 4.**
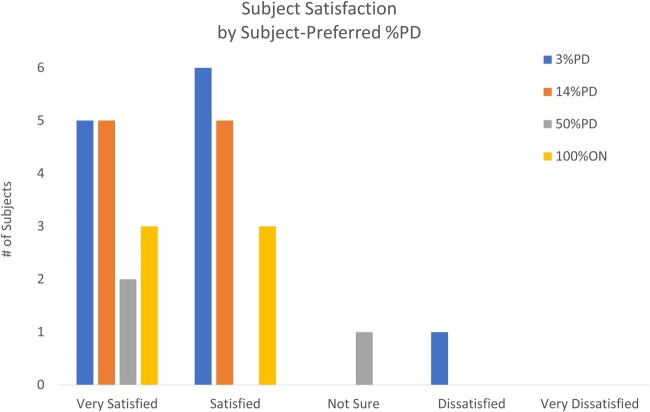
Distribution of satisfaction ratings by subject-preferred %PD during the pulse dose selection period.

All subjects were predominantly satisfied with stimulation (Figure 4), and there was no statistical difference between PD responders (<100%ON) and PD nonresponders (100%ON) (*P* = 1.0) in the satisfaction-grouped ratings during the pulse dose selection period. Additionally, satisfaction did not significantly change for PD responders using <100%ON during the 3-month observational period (*P* = 1.0).

**Figure 5.**
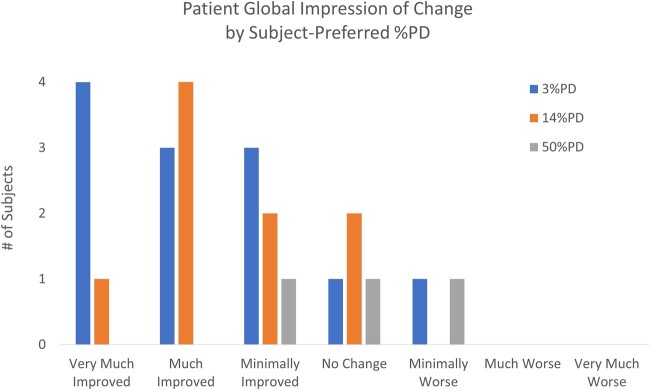
Distribution of PGIC by subject-preferred %PD during the pulse dose selection period.

Similarly, PD responders indicated improvement or no change, relative to the beginning of the study (i.e., using 100%ON), during the pulse dose selection period (see Figure 5). There was no statistical difference between the PGIC results in the pulse dose selection period and the observational period for PD responders (*P* = 0.41).

### Stimulation Program Use and Device Charging

**Figure 6.**
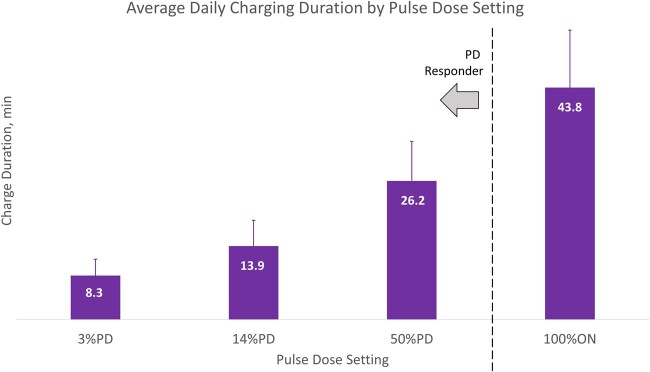
Distribution of average daily charge duration by %PD. (These charge times were provided by subjects who tried these settings, whether preferred or not).

Analysis of IPG log files revealed that 93% of subjects during the pulse dose selection period and 96% of subjects during the observational period used their devices essentially continuously (stimulation was ON for more than 95% of each period). Also, during the pulse dose selection period, stimulation program amplitudes were not significantly different between pulse dose settings: 3%PD = 2.2 ± 0.7 mA, 14%PD = 2.3 ± 0.7 mA, 50%PD = 2.7 ± 0.6 mA, and 100%ON = 2.2 ± 0.7 mA (*P* > 0.05). For device charging, Figure 6 shows the averaged mean daily charge time by %PD. All PD responders as a group charged for an average of 11.3 ± 7.1 minutes per day using their preferred %PD programs. More specifically, average daily charge durations by %PD were as follows: 3%PD = 8.3 ± 3.1 minutes, 14%PD = 13.9 ± 4.9 minutes, 50%PD = 26.2 ± 7.4 minutes, and 100%ON = 43.8 ± 10.9 minutes. Averaged daily charge times for each %PD were significantly different from one another (*H*(3) = 70.2, *P* < 0.001).

**Figure 7.**
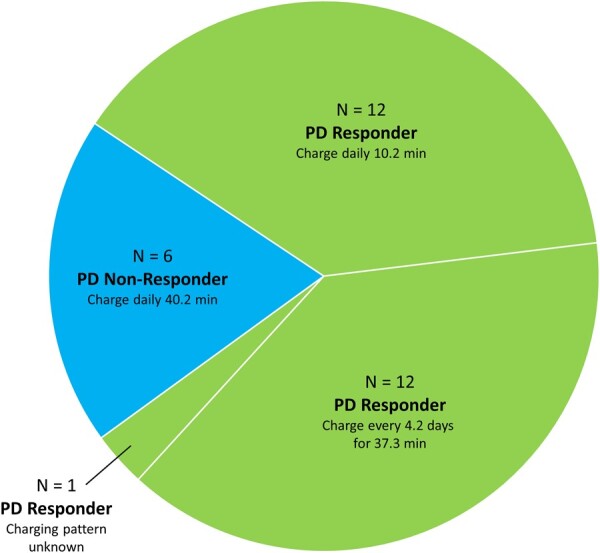
Charging behavior of pulse dose responders and nonresponders.

Figure 7 shows a chart of observed charging behavior. PD nonresponders (n = 6) charged an average of 40.2 ± 12.3 minutes per day. PD responders exhibited two types of behavior: those who charged every day (n = 12) and those who charged less frequently (n = 12). Those PD responders who charged every day averaged a daily charge time of 10.2 ± 4.9 minutes per day, whereas those who charged less frequently (i.e., less than daily) averaged 37.3 ± 30.3 minutes per charge session, performed every 4.2 ± 4.0 days.

### Preference Factors Model

The subjects weighted the three factors (device recharging, pain relief, and “stimulation experience”) on the basis of the strength of their program preference, and the weights were highly correlated with each other, particularly pain relief and stimulation experience, which suggests that the study subjects were equating those two factors. After removal of the “stimulation experience” factor to avoid collinearity, a regression model that includes device charging and pain relief showed that both are significant predictors of program preference (See Table 4).

**Table 4.** Regression analysis of program preference factors

Model Predictors	Coefficient	Std. Err. (Coefficient)	*P* Value
Device recharging	0.184	0.0853	0.04
Pain relief	0.399	0.0796	<0.001

The model coefficients suggest that in the context of preferring a stimulation program, pain relief has approximately twice the value to a patient of device recharging.

## Discussion

To our knowledge, this is the first systematic study of PD with the use of 10-kHz paresthesia-independent SCS. In this prospective observational study, we found that 81% of patients successfully using continuous 10-kHz SCS were PD responders: They could use PD and successfully maintain the low back and leg pain efficacy they had achieved with 100%ON 10-kHz SCS. Earlier anecdotal observations suggested that 67% of subjects would choose 14%PD after successfully using 100%ON, and those observations appeared to be confirmed here [5].

The present study was intended to prospectively and systematically explore a particular programming strategy that was anecdotally observed to be successful in a prior large randomized controlled trial [5, 6]. That randomized controlled trial was designed to be a pragmatic study, and thus it inherently recruited a heterogeneous collection of diagnoses commonly seen in subjects receiving SCS (see Stauss et al. for confirmation of the external validity of Kapural et al.) [10]. The present study was also pragmatic and attempted only to narrow that heterogeneity by requiring that subjects had the diagnosis of failed back surgery syndrome; otherwise, the distribution of diagnoses in the prior randomized controlled trial and the present study align reasonably well. The subgrouping of diagnoses in the present study into different distinct categories for comparison of outcomes by diagnosis is difficult, as many subjects had multiple diagnoses. Conservatively, then, the present study suggests at least that a majority of patients with failed back surgery syndrome do not require 100%ON 10-kHz SCS to achieve good pain relief.

Looking at the distribution of preferred %PD settings, it appears that there is a generally dichotomous response to PD: If a subject is a PD responder, the subject may respond to a very low %PD; otherwise, subjects prefer 100%ON 10-kHz SCS. In our observations, if a subject did not prefer 14%PD, offering a 50%PD setting was not very successful, as only three of nine of these subjects preferred 50%PD to 100%ON. However, subjects who preferred 14%PD had about a 50% chance of responding to 3%PD. Thus, it is clinically reasonable to consider offering a lower %PD if there is a successful response at 14%. This may help to explain why the lowest PD setting we explored, 3%PD, was the dominant mode in the study, initially preferred by 39% of the studied population and maintained by 32% of subjects during the observational period.

Other patient-reported metrics, such as satisfaction and global impression of change, also suggested that pulse-dosed programs provided good outcomes. Twenty-three of the twenty-five (92%) PD responders indicated that they were satisfied or very satisfied with their preferred pulse-dosed program. Seventeen of the twenty-five PD responders (68%) indicated that they experienced improvement with their preferred pulse-dosed program.

A clear clinical benefit of using PD is reduced charging duration. The mean charging duration for PD responders was 11.3 ± 7.1 minutes per day, with those choosing 3%PD charging less than 10 minutes per day. When charging durations were tallied over the course of a week, a PD responder might charge a total of ∼100 minutes. This is 28% less than the typical weekly charging duration for low-frequency paresthesia-based SCS subjects [8]. Despite the “high-frequency” moniker, this challenges the notion that 10-kHz SCS necessarily requires high power; when PD is used, our results suggest it can be more power-efficient than traditional SCS.

In terms of pain intensity, we found no statistical difference in back or leg pain intensity for any %PD setting or 100%ON, despite the significant reduction in the device charging time for <100%ON settings. These results suggest that efficacy is the prime driver of therapy preferences and that patients are willing to use and benefit from PD if it does not negatively affect pain reduction. Indeed, in our preference factors model, we observed that the efficacy of a stimulation program has approximately twice the “decision weight” of reduced charging. This has implications for clinical practice, in that the device chosen for a given subject should assure that efficacious therapy can be delivered with less concern for device recharging.

Lack of a difference in NRS scores between the 100%ON setting and the PD responder’s preferred pulse dose indicates that PD did not improve efficacy. However, it should be noted that the mean NRS scores that subjects reported when they were using their “favorite” 100%ON program at the beginning of the study were 2.1 ± 1.5 and 1.6 ± 1.4 for back and leg pain relief, respectively. These are essentially equivalent to the pain scores of subjects using 100%ON 10-kHz SCS as reported by Kapural et al., representing already excellent outcomes, and thus might not be expected to reach significantly lower values [5].

“Duty-cycled” stimulation has been previously studied for other types of SCS. Intermittently pulsed, low-frequency (e.g., 50-Hz), paresthesia-based SCS has been studied by several groups, using a variety of ON and OFF periods. Kumar et al. reported that patients were given duty-cycled stimulation of 1–2 minutes ON with 5–7 minutes OFF (ostensibly, a range of 14–40% cycling). Notably, they reported challenges in the use of cycled stimulation in subjects who experience paresthesia. If subjects could not abide these longer OFF times, they were switched to cycling parameters with ON/OFF periods that were 1 second or less, and this was reported to improve outcomes. However, if these patients required higher stimulation amplitudes, the cycling generated intolerable tonic muscle activity [11].

Wolter and Winkelmuller explored device use in patients using low-frequency paresthesia-based SCS and found that more than half the subjects they interviewed used some form of intermittent stimulation. In their reporting, ON times were most often >15 minutes (predominantly 30–60 minutes), and the use of cycling and duration of OFF times was generally driven by the pain-free interval the subject experienced after cessation of stimulation. This stimulation OFF distribution was multimodal, with the dominant modes being <15 minutes, 30–60 minutes, and >120 minutes for duration of any effect after cessation of stimulation [12].

Duty cycling of other forms of SCS, such as burst stimulation, have also been reported. Vesper et al. evaluated short ON (5 seconds) and OFF (5, 10 seconds) times for burst stimulation and found no significant differences in pain ratings in these settings compared with continuous-burst SCS delivery [13]. The most preferred mode of intermittent burst SCS delivery was the 50% setting (5 seconds ON, 5 seconds OFF). More recently, Deer et al. explored cycled burst waveforms using a fixed 30-second ON time and varied OFF times: 90, 120, 150, 240, and 360 seconds. They observed a dominant mode of 30 seconds ON and 360 seconds OFF, an 8.3% cycling ratio. This duration of 6 minutes OFF stimulation is in keeping with our results [14].

Short-term evaluation of PD settings provided the primary endpoint for the present study. This meant that subjects evaluated new pulse dose settings for approximately 1–2 weeks. To confirm that these settings were clinically durable, we used a subsequent uncontrolled observational period of 3 months, during which we requested (but did not require) that the subject use the “best” pulse dose program as per their initial preference. Subjects could revert to any %PD at any time during this period. Of those who indicated an initial preference for <100%ON, we found that four subjects (16% of PD responders) decided to return to 100%ON during these 3 months, whereas two other subjects, one PD responder and one PD nonresponder, chose to use predominantly lower %PD settings during the observational period than they initially preferred. Given that initial preference scores showed that 25/31 (81%) of subjects were PD responders and that 87% of PD responders continued with %PD < 100%ON through the observational period, we infer that short-term PD response can reasonably predict longer-term PD responsiveness, where pain relief is maintained while requiring significantly less charge time. Further long-term studies are needed to confirm these results beyond 3 months.

Although no guidance was provided as to charging pattern or behavior during the study, we observed some interesting trends. The reduction in total charge delivered with PD allowed PD subjects to reduce their charging times. Our subjects used this benefit in different ways. Half of PD responders were observed to charge less frequently than once per day during the pulse dose selection period. This group averaged a charging pattern of 37.3 minutes every 4.5 days. The other half of PD responders continued to charge daily; this may have been out of habit (daily charging is recommended for standard 100%ON 10-kHz SCS therapy). The daily charge time in the PD responders was 10.2 minutes, vs 40.2 minutes in the PD nonresponders. PD responders thus gained “charging flexibility.” They could choose between a shorter daily schedule or charging only every few days—whichever suited their lifestyle.

The impact of PD on device lifetime is a complex question. The usable life of rechargeable Li-ion batteries depends on several factors, including battery age, temperature, and state of charge [15]. We observed that PD responders demonstrated a variety of charging patterns, from full recharging every 4+ days to daily recharging for a short period. Overall, PD responders used lower power settings than typical, and, per the Physician’s Manual for the 10-kHz SCS device, the battery may provide service for a longer period of time than the “at least 10 years” of usable battery life [16]. Such predictions, of course, require clinical validation.

### The Concept of “Dosage”

In electrical stimulation, it is tempting to think of stimulation “charge” as the prescribed “dose” [14, 17]. However, this seems an overly simplistic view, without theoretical basis, and might wrongly suggest that *any* of amplitude, pulse width, frequency, duration, or timing of applied stimulation can be chosen to titrate therapy. It is well understood in neurostimulation that specific stimulation parameters provide not only neural activation but also selectivity, defined as the ability to activate one type of neuron vs another type [18, 19]. In paresthesia-based SCS, the adjustment of the stimulation pulse width has been shown to control the activation ratio of larger and smaller dorsal column fibers, which clinically can manifest as paresthesia coverage of different body areas [20, 21]. In paresthesia-free high-kHz SCS, the stimulation frequency has also shown selectivity. Lee et al., in preclinical models of low-intensity-kHz SCS, demonstrated that only 10 kHz and not 1 kHz or 5 kHz could selectively drive GABAergic superficial dorsal horn neurons at clinically-relevant stimulation amplitude levels [22]. Additionally, Al Kaisy et al. have robustly studied a range of kilohertz stimulation frequencies and have demonstrated that pain relief using only 5,882 Hz (vs 1,200 Hz and 3,300 Hz) was significantly improved from sham SCS, even with attempts to equilibrate total charge delivery [23]. Thus, merely calculating the product of stimulation pulse amplitude, width, rate, cycling, etc., is not adequate for assessing a “dosage” of stimulation, especially for different strategies of SCS, such as high kilohertz. Disregarding selectivity of neural stimulation parameters might be compared with ignoring the chirality of two drugs, e.g., s-naproxen, an over-the-counter nonsteroidal anti-inflammatory drug, vs r-naproxen, which, despite having the same chemical formula, is non-analgesic and toxic to the liver [24]. Our finding of different preferred OFF times provides more data for clinical and mechanistic understanding (e.g., duration of therapeutic “washout”), but these results should not be simply bundled into a “charge-per-second” metric. Appropriate clinical studies should be designed to assess whether the concept of “charge as dose” has theoretical validity and therapeutic value.

### Limitations

To focus our efforts and simplify our study design, we did not control amplitude adjustments during application of different %PD settings. Subjects were free to adjust the stimulation amplitudes of their programs to adapt therapy to treat their pain. We observed that there was no significant difference between the stimulation amplitudes for each %PD setting. Optimizing amplitudes for different %PD settings might yield an improvement in outcomes, though we observed that efficacy was statistically similar for all %PD settings and 100%ON.

Additionally, we did not attempt to optimize the spinal location for %PD. All subjects used the same stimulation contacts for all of their %PD program assessments. This essentially fixed the spinal segment and mediolateral position at which we studied the effect of %PD. Ten-kilohertz SCS is believed to have greater rostrocaudal sensitivity than mediolateral sensitivity than traditional, paresthesia-based SCS, so optimization of the longitudinal position of the bipole might have improved outcomes or yielded a different distribution of %PD responders [25]. However, a main focus of the present study was to answer a practical clinical question: Can PD be used successfully in 100%ON 10-kHz SCS responders? To that end, this study suggests that for 81% of such subjects, a simple switch to %PD can maintain efficacy that is already achieved with the use of 100%ON 10-kHz SCS, while reducing charging time.

We studied subjects for only 3 months after the determination of a preferred %PD. Longer-term assessments (e.g., 1 year) would provide better information about how robust such programming is at maintaining pain relief. However, this was a programming study of a device and therapy already shown to be successful in longer-term, higher-level studies [5–7, 10]. Noninvasive changes of programming parameters can be easily performed, and subjects have multiple programs in their devices that they can select from when away from the clinic.

## Conclusions

In this prospective observational study, 81% of 10-kHz SCS responders maintained efficacy and reduced device charging times an average of 64% when using PD. These results suggest that 10-kHz SCS therapy may be successfully used for 10-kHz pulse dose responders with device charging times approximately 30% shorter than traditional SCS. Additionally, subjects gain “battery versatility,” meaning that they can choose a charging regimen that fits their lifestyle. Finally, we observed that patients weighted pain relief as twice as important as device charging, suggesting that efficacy is the critical parameter in the choice of an SCS device.

## Notes

Funding sources: This work was sponsored by Nevro Corp.

Disclosure and conflicts of interest: Dr. Provenzano has consulted for Avanos, Boston Scientific, Esteve, Heron, Medtronic, and Nevro. He has received research support from Avanos, Medtronic, Nevro, Stimgenics, and Abbott. Jordan Tate is a consultant of Nevro. Dr. Mayank Gupta is a consultant for Nevro. Cong Yu has received research support from Boston Scientific and Nevro. Paul Verrills is a consultant to Abbott, Nalu, and Nevro. Maged Guirguis is a consultant to Avanos, Nevro, and Saluda Medical. He has been a Principal Investigator on institutional-funded research to Oschner Health System by Abbott, Avanos, Nalu, Neuros, Nevro, and Saluda. Nathan Harrison has nothing to disclose. Thomas Smith has received support for professional educational activities from Nevro. All the aforementioned authors and/or their research institutions received support for this study. Rose Azalde and Kerry Bradley are full-time employees and shareholders of Nevro Corp.

Trial registration: ISRCTN Registry number 54708653.
